# The N-terminal region of p27 inhibits HIF-1*α* protein translation in ribosomal protein S6-dependent manner by regulating PHLPP-Ras-ERK-p90RSK axis

**DOI:** 10.1038/cddis.2014.496

**Published:** 2014-11-20

**Authors:** D Zhang, J Liu, X Mi, Y Liang, J Li, C Huang

**Affiliations:** 1 Nelson Institute of Environmental Medicine, New York University School of Medicine, 57 Old Forge Road, Tuxedo, NY 10987, USA

## Abstract

P27 was identified as a tumor suppressor nearly two decades, being implicated in cell-cycle control, differentiation, senescence, apoptosis and motility. Our present study, for the first time to the best of our knowledge, revealed a potential role of p27 in inhibiting S6-mediated hypoxia-inducible factor-1*α* (HIF-1*α*) protein translation, which contributed to the protection from environmental carcinogen (sodium arsenite)-induced cell transformation. Our findings showed that depletion of p27 expression by knockout and knockdown approaches efficiently enhanced S6 phosphorylation in arsenite response via overactivating Ras/Raf/MEK/ERK pathway, which consequently resulted in the stimulation of p90RSK (90 kDa ribosomal S6 kinase), a direct kinase for S6 phosphorylation. Although PI3K/AKT pathway was also involved in S6 activation, blocking AKT and p70S6K activation did not attenuate arsenite-induced S6 activation in p27−/− cells, suggesting p27 specifically targeted Ras/ERK pathway rather than PI3K/AKT pathway for inhibition of S6 activation in response to arsenite exposure. Further functional studies found that p27 had a negative role in cell transformation induced by chronic low-dose arsentie exposure. Mechanistic investigations showed that HIF-1*α* translation was upregulated in p27-deficient cells in an S6 phosphorylation-dependent manner and functioned as a driving force in arsenite-induced cell transformation. Knockdown of HIF-1*α* efficiently reversed arsenite-induced cell transformation in p27-depleted cells. Taken together, our findings provided strong evidence showing that by targeting Ras/ERK pathway, p27 provided a negative control over HIF-1*α* protein synthesis in an S6-dependent manner, and abrogated arsenite-induced cell transformation via downregulation of HIF-1*α* translation.

Numerous physiological, pathological and pharmacological stimuli have been reported to cause S6 phosphorylation, which results in the regulation of global protein synthesis, cell growth, proliferation and glucose homeostasis.^1^ The phosphorylation sites of mammals S6 have been mapped to the C-terminal region at five clustered serine residues, for example, S235/236/240/244/247, which are evolutionarily conserved in higher eukaryotes.^2, 3, 4, 5, 6, 7, 8, 9, 10^ Clinically hyperphosphorylation of S6 is frequently observed in human malignancies, such as breast cancer,^11^ sarcoma^12^ and acute leukemia.^13^ Increased phosphorylation of S6, along with increased phosphorylation of translation initiation factor 4E-binding protein 1, increased expression of eukaryotic elongation factor 2 kinase and decreased expression of programmed cell death protein 4 have been categorized as four major aberrations of the translation process implicated in breast cancer when predicting overall survival or recurrence-free survival of patients.^11, 14, 15^ Therefore, targeting S6 phosphorylation and its related signaling pathway is a conventional strategy implicated in therapeutic intervention of human cancers.^16^ However, the findings obtained from S6^P−/−^ knock-in mice, which contained replacement of all five serine residues with alanines, have complicated our understandings about the role of S6 phosphorylation in protein synthesis.^17^ Contradictory to the previous observations, defects in S6 phosphorylation even increased global protein synthesis rate.^17^ One suggested mechanism is that S6 phosphorylation might specifically regulate each step of protein translation,^18^ namely S6 phosphorylation might finely upregulate translation initiation, while downregulate other critical steps of translation, such as elongation and termination.^18^ It is also likely that the effect of S6 phosphorylation on protein translation is gene-specific, depending on the individual transcript stereoscopic structure or depending on the type of initiation steps, such as cap- or IRES-mediated translation initiation. Further investigations to discriminate these possibilities will help envision the exact function of S6 phosphorylation.

P27 is initially identified as a potent negative cell-cycle regulator that preferentially binds to and inhibits cyclin D–CDK4/6 and cyclin E/A–CDK2 complexes.^19, 20^ Later, more in-depth studies indicate that p27 is a multifunctional protein that exerts additional activities on apoptosis, cell adhesion and migration, independent of its cyclin/CDK binding and inhibition properties.^19, 21, 22, 23, 24, 25^ Our present study, for the first time to the best of our knowledge, revealed a potential role of p27 in inhibiting *hif-1a* mRNA translation via regulating Ras/Raf/MEK/ERK pathway that was responsible for the stimulation of p90RSK (90 kDa ribosomal S6 kinase), the direct kinase for S6 phosphorylation. Further functional studies demonstrated that through inhibiting S6-mediated hypoxia-inducible factor-1*α* (HIF-1*α*) translation, p27 contributed to the protection from environmental carcinogen (sodium arsenite)-induced cell transformation. Therefore, from a novel point of view, our findings provide one more piece of strong evidence for the suppressive role of p27 in the process of tumor promotion and progression in arsenic response, which is achieved by inhibition of HIF-1*α* protein translation.

## Results

### P27 inhibited arsenite-induced ribosomal protein S6 phosphorylation

**Figure 1:**
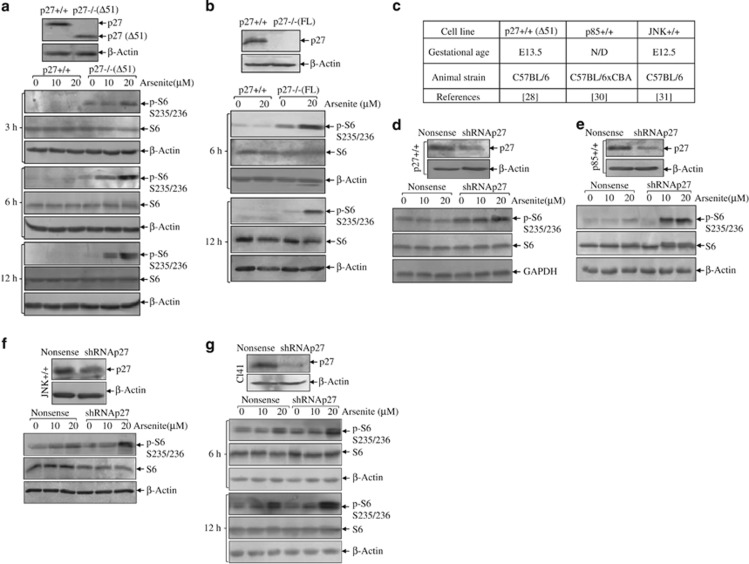
P27 inhibits arsenite-induced S6 phosphorylation. (**a** and **b**) Two pairs of p27-knockout cells, including p27−/−(Δ51) (**a**) and p27−/−(FL) (**b**) and their respective wild-type cells were seeded into 6-well plates and cultured overnight in the normal medium containing 10% FBS. Then, the cells were starved for 36 h in the fresh medium with 0.1% FBS. Later, the cells were then exposed to 10 and 20 *μ*M arsenite for the indicated time periods in the fresh medium with 0.1% FBS . Western blotting assay was carried out for the detection of total and phospho-S6. *β*-Actin was used as a loading control. (**c**–**f**) Wild-type MEFs generated from different mouse strain backgrounds and gestational ages as summarized (**c**) were stably transfected with short hairpin RNA (shRNA) p27. The stable transfectants of shRNA p27 and the nonsense controls were treated with arsenite as indicated. Western blotting assay was carried out to compare S6 phosphorylation (**d**–**f**). (**g**) Mouse epidermal Cl41 cells were stably transfected with shRNA p27 to detect S6 phosphorylation after arsenite treatment

Mouse embryonic fibroblast (MEF) is a relatively less differentiated mesodermally derived cell type, so that most genes are actively transcribed and translated in MEF. Therefore, MEF is widely used in the studies of differentiation, transformation, senescence and apoptosis.^26, 27^ We used two pairs of p27−/− MEFs and their littermated p27+/+ MEFs to minimize the off-target effects generated during cell line establishment, one being named as p27−/−(Δ51)^28^ because of the deletion of N-terminal 51 amino acids, and the other named as p27−/−(FL) because it harbored deletion of the entire coding region^29^ (Figures 1a and b, top panels). Using these cells, we found that arsenite treatment caused a strong induction of phospho-S6 at S235/236 in p27−/− cells compared with that in p27+/+ cells at all the time points tested (3–12 h), regardless of the knockout methods used (Figures 1a and b, bottom panels), indicating that p27 might abrogate arsenite-induced S6 phosphorylation at S235/236. Elevations of phospho-S6 at S235/236 were also observed in the stable transfectants of shRNA p27 generated from MEFs of different backgrounds and gestational ages,^28, 30, 31^ (Figures 1c–f) as well as from mouse epidermal Cl41 cells (Figure 1g), indicating that the inhibitory effect of p27 on arsenite-induced S6 phosphorylation at S235/236 was prevalent, which was neither cell type-specific nor developmental stage-dependent.

**Figure 2:**
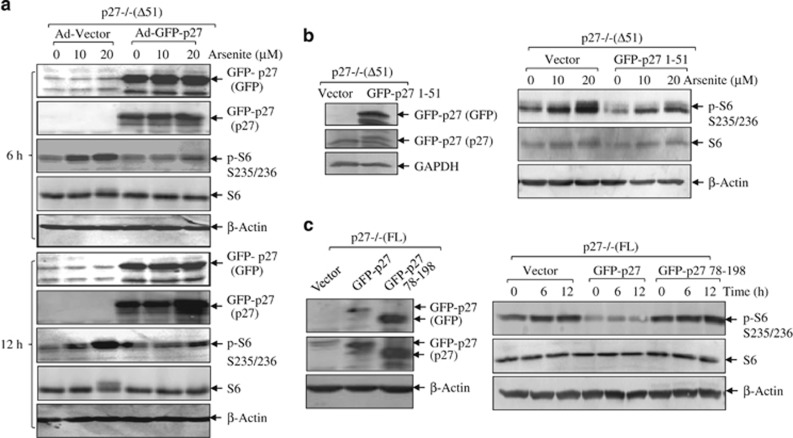
N terminus of p27 is responsible for its inhibitory effect on S6 phosphorylation. (**a**) GFP-tagged p27 was introduced into p27−/−(Δ51) cells by adenovirus delivery system. Twenty-four hours later, the above cells were treated with arsenite for 6 and 12 h. Western blotting assay was then carried out to detect S6 phosphorylations. The stable cell lines were identified by anti-GFP and p27 antibodies. (**b**) GFP-tagged N-terminal region of human p27 (aa 1–51) was stably transfected into p27−/−(Δ51) cells. The transfection efficiency was detected using anti-GFP and p27 antibodies. The stable transfectant of GFP-p27 (aa 1–51) and the vector controls were treated with arsenite as indicated. Western blotting assay was carried out to detect S6 phosphorylation. (**c**) GFP-tagged full-length human p27 or the C-terminal region (aa 78–198) was stably transfected into p27−/−(FL) cells. The stable cell lines were identified using anti-GFP and p27 antibodies. After arsenite treatment, the above cells were lysed and subjected to western blotting for S6 phosphorylation

Our findings were further enforced by reconstitution experiment, in which either the full-length human p27 or the N-terminal region (aa 1–51) of p27 that was fused to 3′ GFP-tag was delivered into p27−/−(Δ51) cells by adenovirus (Figure 2a) or stable transfection (Figure 2b). In both cases, an obvious inhibition of arsenite-induced phospho-S6 at S235/236 was observed (Figures 2a and b). To verify that the remaining C-terminal portion of p27 (aa 52–197) in p27−/−(Δ51) cells was not able to fulfill the function of full-length p27, we stably transfected the full-length GFP-tagged p27 and C terminus (aa 78–198) of p27 into p27−/−(FL) cells, respectively. As shown in Figure 2c, the expression of full-length p27 was able to completely abrogate induction of phospho-S6 by arsenite, whereas transfection of C terminus of p27 (aa 78–198) did not render the repressive effect, indicating that the N terminus of p27 (aa 1–51) was critical for its inhibitory effect on S6 phosphorylation induced by arsenite.

### AKT/p70S6K pathway was not responsible for hyperphosphorylation of S6 caused by p27 depletion

**Figure 3:**
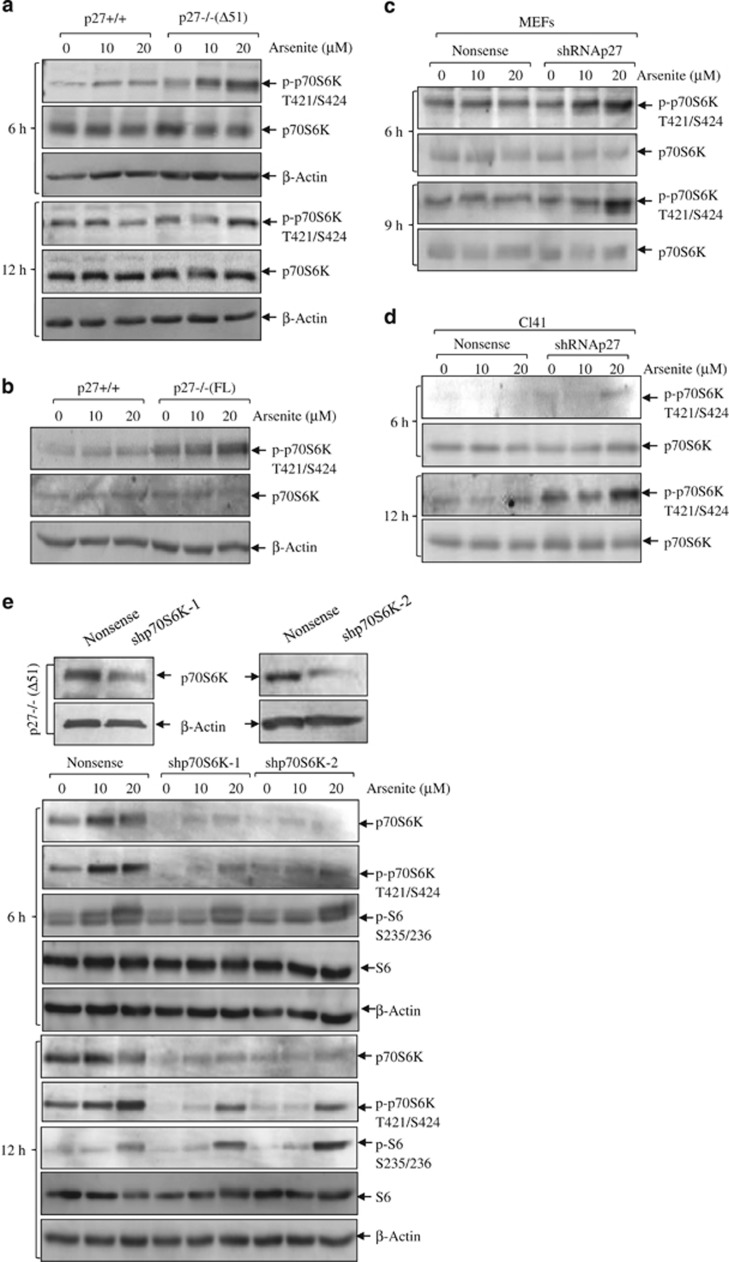
The inhibitory effect of p27 on S6 phosphorylation is not due to p70S6K. P27−/−(Δ51) (**a**) and p27−/−(FL) cells (**b**) and their respective wild-type cells were seeded into 6-well plates and cultured overnight in the normal medium containing 10% FBS. Then, the cells were starved for 36 h in the fresh medium with 0.1% FBS. Later, the cells were exposed to 10 and 20 *μ*M arsenite for the indicated time periods (**a**) or 6 h (**b**) in the fresh medium with 0.1% FBS. Western blotting assay was carried out for the detection of total and phospho-S6. *β*-Actin was used as a loading control. (**c** and **d**) Short hairpin RNA (shRNA) p27 stable transfectants of wild-type MEFs (**c**) and Cl41 (**d**) were treated with 10 and 20 *μ*M arsenite for the indicated time periods. The cells were lysed and subjected to western blotting assay for the detection of total and phospho-p70S6K. (**e**) Two sets of shRNA against mouse p70S6K were transfected into p27−/−(Δ51) cells and the stable transfectants were identified using anti-p70S6K antibody. The indicated cells were exposed to arsenite and activation of p70S6K and S6 was assessed by western blotting assay

We examined activation of p70S6K using phospho-T421/S424 as an indicative marker. As shown in Figures 3a and b, the phosphorylations of p70S6K on T421/S424 induced by arsenite were elevated in p27−/− cells compared with those in p27+/+ cells at all the time points examined in both pairs of knockout cells. The findings were further reproduced in shRNA p27 transfectants of both MEFs and Cl41 cells (Figures 3c and d), suggesting that arsenite-induced activation of p70S6K was upregulated upon p27 depletion. Next, to determine the involvement of p70S6K in the activation of S6 in p27-depleted cells, we stably transfected two sets of shRNA against p70S6K into p27−/−(Δ51) cells (Figure 3e, upper panels) and checked S6 phosphorylation induced by arsenite. As shown in Figure 3e (lower panels), in both p70S6K-knockdown transfectants arsenite-induced phosphorylation of p70S6K was apparently reduced but not that of S6, indicating that p70S6K was not the upstream kinase that was responsible for the elevated S6 phosphorylation in p27-depleted cells in response to arsenite.

**Figure 4:**
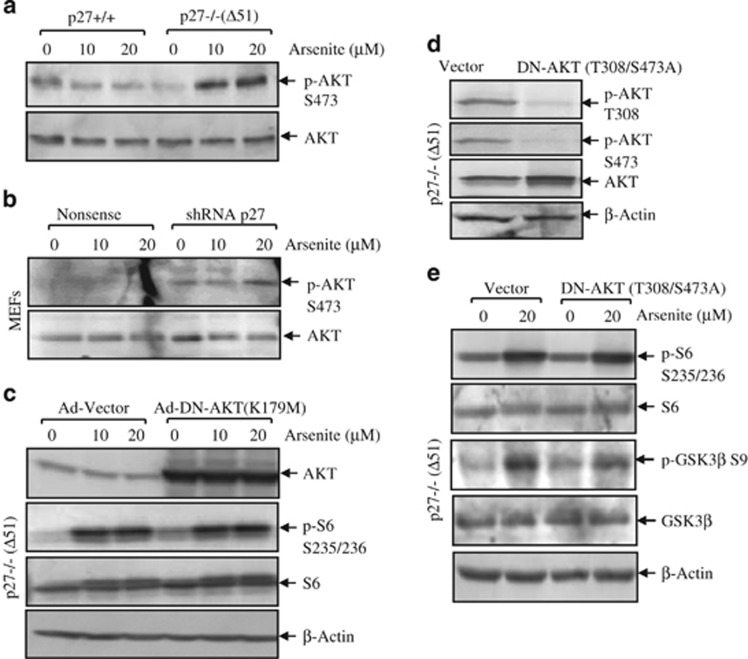
AKT is not required for the inhibitory effect of p27 on S6 phosphorylation. P27−/−(Δ51) and p27+/+ cells (**a**) as well as short hairpin RNA (shRNA) p27 and nonsense controls of wild-type MEFs (**b**) were treated with 10 and 20 *μ*M arsenite for 6 h. The cells were lysed and subjected to western blotting assay for the detection of total and phospho-AKT. (**c**) Dominant-negative mutant of AKT (K179M) was introduced into p27−/−(Δ51) cells by adenovirus delivery system. The cells were treated with 20 *μ*M arsenite for 6 h as indicated. The cells were lysed and subjected to western blotting assay for S6 phosphorylation. The infection efficiency was detected by anti-AKT antibody. (**d** and **e**) The plasmid containing AKT mutations at T308/S473A was stably transfected into p27−/−(Δ51) cells. The stable transfectants were identified by anti-phospho-AKT T308 and S473 antibodies (**d**). The cells were treated with 20 *μ*M arsenite for 6 h as indicated. The cells were lysed and subjected to western blotting assay for S6 phosphorylation (**e**)

In our current studies, we found that arsenite-induced phospho-AKT S473 was elevated in p27−/− cells and shRNA p27 transfectants when compared with that in wild-type and nonsense control cells, respectively (Figures 4a and b). To provide direct evidence for the involvement of AKT in the activation of S6, adenovirus carrying dominant-negative form of AKT, K197M, was used to infect p27−/−(Δ51) cells. The infection efficiency was demonstrated by the remarkable elevation of AKT expression (Figure 4c). However, ectopic expression of Ad-DN-AKT (K197M) in p27−/−(Δ51) cells failed to reduce phosphorylation of S6, indicating that AKT was not required for the activation of S6 in p27−/−(Δ51) cells in arsneite response. Consistently, stable transfection of another dominant mutant form of AKT, T308/S473A, into p27−/−(Δ51) cells (Figure 4d) was not able to decrease S6 phosphorylation (Figure 4e), but it could partially reduce phosphorylation of GSK3*β* at S9, a well-known downstream target of AKT (Figure 4e). Therefore, our results suggested that AKT might not be the upstream kinase responsible for S6 phosphorylation in arsenite response in p27-depleted cells.

### P27 regulated arsenite-induced S6 phosphorylation via ERK/p90RSK pathway

**Figure 5:**
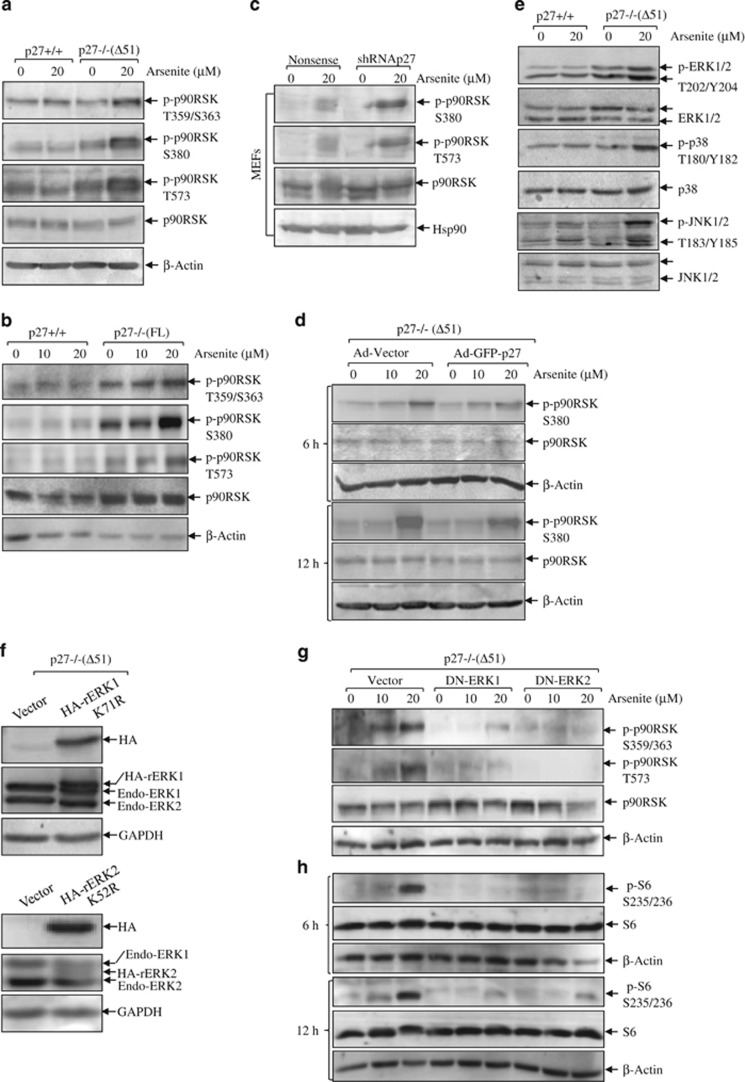
ERK/p90RSK pathway is involved in S6 phosphorylation in arsenite response in p27KO cells. P27−/−(Δ51) (**a**) and p27−/−(FL) cells (**b**) and their respective wild-type cells were seeded into 6-well plates and cultured overnight in the normal medium containing 10% FBS. Then, the cells were starved for 36 h in the fresh medium with 0.1% FBS. Later, the cells were exposed to arsenite for 6 h in the fresh medium with 0.1% FBS. The cells were lysed and subjected to western blotting assay for the detection of p90RSK activation. (**c**) short hairpin RNA (shRNA) p27 and nonsense controls of wild-type MEFs were treated with 20 *μ*M arsenite for 6 h. The cells were lysed and subjected to western blotting assay for detection of p90RSK activation. (**d**) GFP-tagged p27 was introduced into p27−/−(Δ51) cells by adenovirus delivery system. The cells were treated with 20 *μ*M arsenite for 6 and 12 h as indicated. The cells were lysed and subjected to western blotting assay for the detection of p90RSK activation. (**e**) P27+/+ and p27−/−(Δ51) cells were treated with arsenite and then subjected to western blotting assay for the detection of MAPK pathway activation. (**f**–**h**) Dominant-negative ERK1/2 was stably transfected into p27−/−(Δ51) cells. The stable transfectants were identified by overexpression of hemagglutinin (HA)-tagged ERK K72/52R (**f**). The activation of p90RSK/S6 was assessed by western blotting assay (**g** and **h**)

We checked p90RSK activation and found that the phosphorylations of p90RSK at T359/S363, S380 and T573 were elevated in p27−/−(Δ51) cells compared with those in p27+/+ cells (Figure 5a). Similar findings were observed in p27−/−(FL) and shRNA p27 transfectants when compared with their wild-type and nonsense control cells (Figures 5b and c). In addition, introduction of GFP-p27 partially blocked the elevated phoshporylation of p90RSK in p27−/−(Δ51) cells (Figure 5d). MEK/ERK pathway is known as the upstream activators responsible for p90RSK-mediated S6 phosphorylation,^1^ thus in turn we checked ERK activation as well as the other two kinases of MAPK family. As shown in Figure 5e, increment in the activation of all the three MAPKs was observed in p27−/−(Δ51) cells compared with those in p27+/+ cells following arsenite treatment. To further verify the involvement of ERK in S6 phosphorylation in p27−/− cells, we transfected DN-ERK1/2 into p27−/−(Δ51) cells. The identification of stable transfectants of DN-ERK1/2 was shown by overexpression of HA and ERK1/2 (Figure 5f). Overexpression of either DN-ERK1 or DN-ERK2 showed an overt inhibition on p90RSK phosphorylation (Figure 5g) and S6 activation (Figure 5h). Therefore, we concluded that ERK/p90RSK pathway participated in S6 phosphorylation induced by arsenite in p27-depleted cells.

### P27 inhibited activation of Ras/Raf/MEK pathway

**Figure 6:**
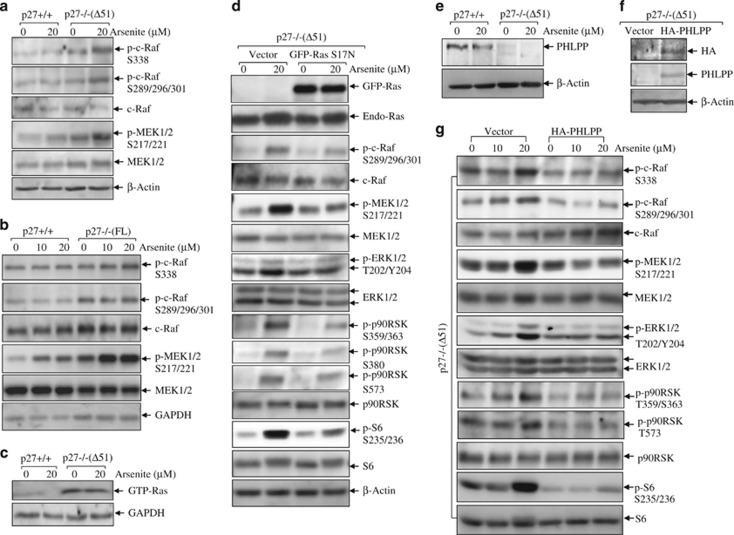
P27 maintains PHLPP expression, which inhibits Ras/Raf/MEK/ERK/ p90RSK/S6 cascade. P27−/−(Δ51) (**a**) and p27−/−(FL) cells (**b**) and their respective wild-type cells were seeded into 6-well plates and cultured overnight in the normal medium containing 10% FBS. Then, the cells were starved for 36 h in the fresh medium with 0.1% FBS. Later, the cells were exposed to arsenite for 6 h in the fresh medium with 0.1% FBS. The cells were lysed and subjected to western blotting assay to detect activation of Raf/MEK pathway. (**c**) Ras activity was detected in p27+/+ and p27−/−(Δ51) cells with or without 20 *μ*M arsenite treatment for 3 h. (**d**) GFP-HRas S17N was transfected into p27−/−(Δ51) cells and its effects on Raf/MEK/ERK/p90RSK/S6 activation was detected by western blotting assay. (**e**) PHLPP expression was detected in p27+/+ and p27−/−(Δ51) cells by western blotting assay. (**f** and **g**) HA-PHLPP was transfected into p27−/−(Δ51) cells. The stable transfectants were identified by HA-tag and overexpression of PHLPP (**f**). The indicated transfectants were treated with 20 *μ*M arsenite for 6 h. The Raf/MEK/ERK cascade activation was detected (**g**)

To determine how p27 inhibits ERK activation in arsenite response, the activation of upstream kinases, the Raf/MEK cascade, was compared between p27+/+ and p27−/− cells. As shown in Figure 6a, phosphorylations of c-Raf at S289/296/301 and S338, the central points for controlling Raf activation, were found substantially upregulated by arsenite in p27−/−(Δ51) cells compared with those in p27+/+ cells. An identical increasing pattern was found on phosphorylations of MEK1/2 at S217/221 in p27−/−(Δ51) cells (Figure 6a). Then, we further extended these findings to p27−/−(FL) cells (Figure 6b). In addition, we performed Ras activity assay and found that in p27−/−(Δ51) cells Ras was kept in constitutively activated GTP-bound form (Figure 6c), suggesting that p27 might target Ras for deactivation of the downstream kinases. To prove this, we transfected GFP-tagged dominant-negative form of Ras, S17N, into p27−/−(Δ51) cells. Our results showed that overexpression of Ras S17N efficiently blocked activation of Raf/MEK/ERK cascade, further leading to the impairment of p90RKS and S6 phosphorylations (Figure 6d). Thus, the results demonstrated that by deactivating Ras/Raf/MEK/ERK/p90RSK pathway, p27 inhibited arsenite-induced S6 phosphorylation.

### P27 maintained PHLPP expression and promoted its inhibitory activity toward Ras/ERK/p90RSK cascade

As a member of the small GTPase family, Ras oscillates between an inactive GDP-bound state in the cytosol and an active GTP-bound form in the plasma membrane.^32^ Recent studies suggest that pleckstrin homology domain leucine-rich repeat protein phosphatase (PHLPP) has an important role in regulating Ras activation by interacting with guanine nucleotide-free form of Ras, hampering nucleotide binding for Ras activation, thereby negatively modulating downstream ERK pathway activation.^32, 33^ Therefore, we checked the expression of PHLPP in p27+/+ and p27−/−(Δ51) cells. As shown in Figure 6e, the basal level of PHLPP was depleted in p27−/−(Δ51) cells, and arsenite treatment decreased PHLPP1 expression in p27+/+ cells. To verify whether the impaired expression of PHLPP in p27−/−(Δ51) cells led to increment in Ras/ERK/S6 pathway activation, we stably transfected HA-PHLPP into p27−/−(Δ51) cells, and the transfectants were identified by overexpression of HA and PHLPP (Figure 6f). The ectopic expression of HA-PHLPP markedly reduced the activation of Raf/MEK/ERK/p90RSK/S6 cascade (Figure 6g). Therefore, we concluded that PHLPP expression was maintained by p27 and mediated the deactivation of Ras/ERK/S6 pathway in arsenite response.

### P27 inhibited HIF-1*α* translation because of arsenite exposure

**Figure 7:**
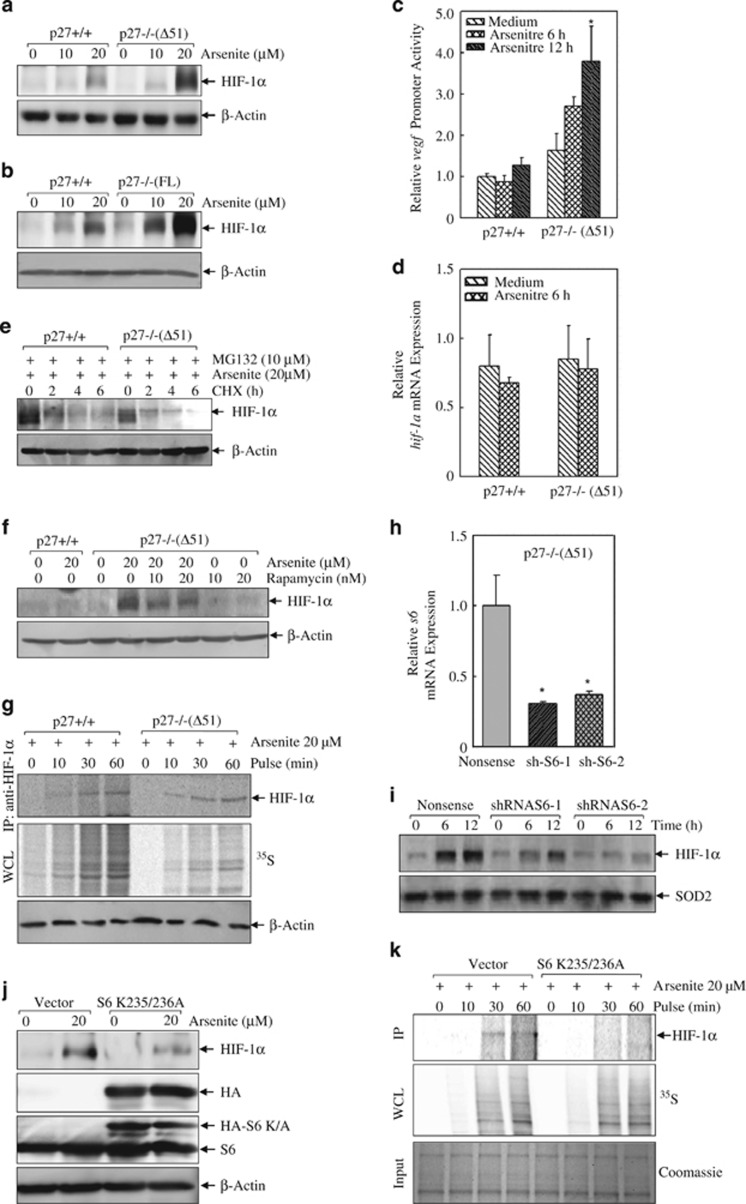
P27 represses HIF-1*α* induction by arsenite at the translational level. P27−/−(Δ51) (**a**) and p27−/−(FL) cells (**b**) and their respective wild-type cells were seeded into 6-well plates and cultured overnight in the normal medium containing 10% FBS. Then, the cells were starved for 36 h in the fresh medium with 0.1% FBS. Later, the cells were exposed to arsenite for 12 h in the fresh medium with 0.1% FBS. The cells were lysed and subjected to western blotting assay for the detection of HIF-1*α* induction. (**c**) Transcription of *vegf* was compared between p27+/+ and p27−/−(Δ51) cells using luciferase reporter assay following 20 *μ*M arsenite treatment for 6 h and 12 h. (**d**) P27+/+ and p27−/−(Δ51) cells were treated with 20 *μ*M arsenite for 6 h. *hif-1a* mRNA expression was detected by real-time PCR. (**e**) P27+/+ and p27−/−(Δ51) cells were pretreated with 10 *μ*M MG132 and 20 *μ*M arsenite for 6 h and exposed to 50 *μ*g/ml CHX for 2, 4 and 6 h after the removal of MG132 and arsenite. Levels of HIF-1*α* were detected by western blotting assay. (**f**) P27+/+ and p27−/−(Δ51) cells were treated with 20 *μ*M arsenite in the absence or presence of 10 and 20 nM rapamycin for 12 h. HIF-1*α* induction was detected by western blotting. (**g**) Cells were exposed to 20 *μ*M arsenite for 9 h. Newly synthesized HIF-1*α* protein was monitored by pulse labeling assay. (**h**) Two sets of short hairpin RNA (shRNA) against mouse S6 were stably transfected into p27−/−(Δ51) cells, and the knockdown efficiency was identified using real-time PCR. (**i**) The stable transfectants of S6 shRNA and nonsense control cells were treated with 20 *μ*M arsenite for 6h and 12 h. The cells were lysed and subjected to western blotting assay to detect HIF-1*α* induction. (**j** and **k**) HA-S6 K235/236A plasmid was transfected into p27−/−(Δ51) cells. HIF-1*α* induction by arsenite was assessed by western blotting (**j**) and protein synthesis was detected by pulse labeling assay after arsenite treatment for 9 h (**k**)

To investigate whether arsenite could affect HIF-1*α* protein translation and whether inhibition of Raf/ERK/S6 pathway by p27 would attenuate it, we first checked HIF-1*α* induction by arsenite in p27+/+ and p27−/−(Δ51) cells. As shown in Figure 7a, compared with the mild induction of HIF-1*α* in p27+/+ cells, a remarked accumulation of HIF-1*α* protein was observed in p27−/−(Δ51) cells. The inhibition of HIF-1*α* induction by p27 was further extended in p27−/−(FL) and p27+/+ cells (Figure 7b). In addition, the transcription of *vegf*, a well-defined downstream target of HIF-1*α*, was also found upregulated in p27−/−(Δ51) cells in response to arsenite treatment (Figure 7c). While the real-time PCR result clearly indicated that the mRNA level of *hif-1α* was not upregulated in p27−/−(Δ51) cells (Figure 7d), suggesting that p27 might regulate HIF-1*α* induction by arsenite through a translational or posttranslational mechanism. Considering our previous work showing that arsenite was involved in prevention of HIF-1*α* protein degradation, we checked the protein turnover rate of HIF-1*α* using cycloheximide (CHX), a protein synthesis inhibitor, to block the *de novo* production of proteins. As shown in Figure 7e, HIF-1*α* protein was firstly accumulated by treatment of 20 *μ*M arsenite plus 10 *μ*M MG132 for 6 h in both p27+/+ and p27−/−(Δ51) cells. After removal of arsenite and MG132, HIF-1*α* protein began to degrade in the presence of CHX at a comparable rate in these two cell lines (Figure 7e), suggesting that p27 did not alter the protein turnover process of HIF-1*α*. Subsequently, we used rapamycin to interfere with mTORC1 activity and found that it reduced HIF-1*α* induction in p27−/−(Δ51) cells (Figure 7f), indicating that p27 inhibited HIF-1*α* translation. To this end, short-term pulse-labeling assay was used to examine HIF-1*α* protein translational process in both p27+/+ and p27−/−(Δ51) cells. As shown in Figure 7g, the incorporation of ^35^S-methionine/cysteine into newly synthesized HIF-1*α* protein was gradually increased along with the isotope incubation time in both p27+/+ and p27−/−(Δ51) cells. By normalizing over the global protein synthesis rate, the HIF-1*α* protein synthesis rate was much higher in p27−/−(Δ51) cells than that in p27+/+ cells, indicating that p27 interfered with HIF-1*α* protein translation upon arsenite exposure. We further knocked down S6 expression by two sets of shRNAs in p27−/−(Δ51) cells (Figure 7h), and found that HIF-1*α* protein induction by arsenite was reduced accordingly (Figure 7i). Considering the fact that S6, unlike its phosphorylation, is a critical protein for functional ribosomes so that its complete absence or the lack of even just one allele leads to early embryonic lethality^34^ or to blockage of T-cell proliferation,^17^ knockdown of S6 may only suggest that HIF-1*α* synthesis is highly sensitive to the compromised ribosome activity. To show explicitly the role of S6 phosphorylation in HIF-1*α* protein translation under the control of the p27 signaling in arsenite response, we introduced S6 S235/236A double mutant, which specifically abolished phosphorylation of Ser235/236 in endogenous S6.^18^ As shown in Figure 7j, ectopic expression of HA-S6 S235/236A in p27−/− (Δ51) cells efficiently attenuated arsenite-induced HIF-1*α* upregulation. In line with this, HIF-1*α* protein synthesis detected by pulse-labeling assay was markedly repressed by the introduction of HA-S6 S235/236A in p27−/−(Δ51) cells (Figure 7k), suggesting that p27 inhibited HIF-1*α* translation in an S6-phosphorylation-dependent manner in arsenite response.

### P27 prevented chronic arsenite-induced cell transformation in an HIF-1*α*-dependent manner

**Figure 8:**
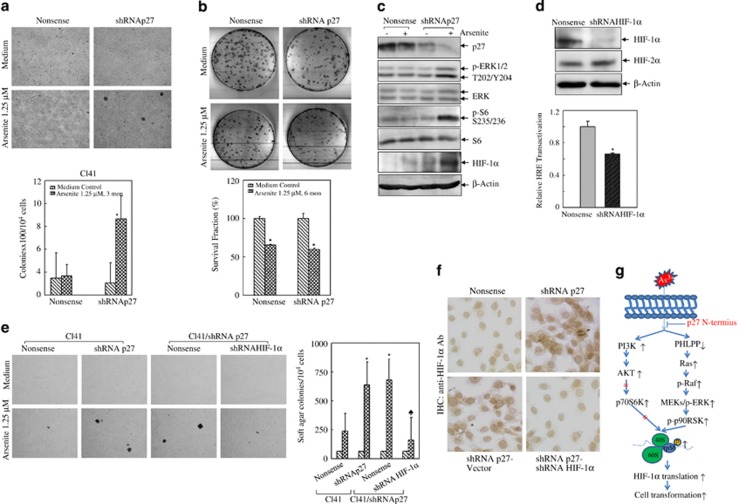
P27 suppresses low-dose arsenite-induced cell transformation. (**a**) shRNA p27 and control transfectants of Cl41 cells were repetitively exposed to 1.25 *μ*M arsenite for 3 months to induce cell transformation, which was assessed by soft agar assay. (**b**) The above cells were plated in triplicate into 100-mm culture dish at the density of 500 cells per dish and cultured for 2 weeks. Cells were fixed by ice-cold methanol and stained with Giemsa solution The symbol (*) indicates a significant difference in survival fraction of medium control and between arsenite treatment (*P*<0.01). The value was showed as mean±S.D. from three independent experiments. (**c**) The protein expression profiles of chronic arsenite-treated cells were subjected to western blotting assay. (**d** and **e**) shRNA HIF-1*α* was stably transfected into shRNA p27 cells and identified using anti-HIF-1*α* antibody as well as HRE-dependent luciferase activity (**d**). Cell transformation capabilities of the indicated cells were compared using soft agar assay (**e**). (**f**) Immunohistochemistry analysis of HIF-1*α* expression in arsenite chronically exposed Cl41 nonsense, shRNA p27, shRNA p27-Vector and shRNA p27-shRNA HIF-1*α* cells. (**g**) The scheme showing how p27 inhibits S6 activation and its biological consequence

Mouse epidermal Cl41 model is a well-characterized model for neoplastic transformation in response to tumor promoters. Distinct from several other rodent cells in culture, which undergo spontaneous neoplasmic transformation because of chromosomal aberrations, the Cl41 model is stably free of spontaneous transformation and responsive to tumor promoters. Using this cell model, our previous work successfully demonstrates that chronic exposure to environmental relevant low-dose of arsenite induces cell transformation *in vitro*.^35^ Stable transfectants of Cl41 nonsense control and shRNA p27 were subjected to repetitive arsenite treatment at 1.25 *μ*M. Soft agar assays were carried out monthly to trace phenotypic change of anchorage-independent cell growth, which was used as an indicator of cell transformation. As shown in Figure 8a, when arsenite exposure was accumulated to 3 months, the remarkable appearance of colonies was observed in shRNA p27 transfectants in soft agar, whereas no detectable colonies were formed in control transfectants as expected since our previous work showed that in parental Cl41 cells it took up to 6 months to induce cell transformation by 1.25 *μ*M arsenite exposure.^35^ As p27 is known to regulate cell proliferation and apoptosis, we carried out clonogenic assays to quantify the overall rates of cell growth. As shown in Figure 8b, chronic arsenite treatment readily led to significant reduction in cell viability in both Cl41 nonsense and shRNA p27 cells at comparable rates (65.30±0.9% *versus* 59.63±1.8%), indicating that the upregulation of cell transformation observed in Cl41 shRNA p27 cells might not be caused by proliferative factors. We further found that the major pathways that were identified to be deactivated by p27 in acute arsenite exposure were reproduced in the chronic arsenite exposure. As shown in Figure 8c, upregulation of ERK/S6 phosphorylation and HIF-1*α* induction by arsenite were observed in shRNA p27 transfectants as compared with those in control transfectants. To further verify whether HIF-1*α* was the driving force of chronic arsenite-induced cell transformation in the absence of p27, we knocked down HIF-1*α* expression in shRNA p27 cells. As shown in Figure 8d, introduction of shRNA HIF-1*α* markedly reduced HIF-1*α* protein expression as well as hypoxia-responsive element (HRE) reporter luciferase activity, which reflected HIF-1*α*-dependent transcription factor activity. HRE has a core binding consensus of RCGTG (R: A/G), which is conserved in more than one hundred hypoxia-responsive genes and also has strong affinity toward HIF-1*α* and HIF-2*α*.^36^ Therefore, we further checked whether there was any interaction between these two isoforms. We found that knockdown HIF-1*α* marginally affected HIF-2*α* expression, indicating that interfering HIF-1*α* had little effects on HIF-2*α* activity (Figure 8d). Soft agar assay results showed that more colonies were found in shRNA p27 cells compared with those of control cells after chronic arsenite treatment for 6 months (Figure 8e), which was consistent with what we observed in 3-month arsenite treatment cells (Figure 8a). More importantly, knockdown of HIF-1*α* efficiently reversed cell transformation of shRNA p27 cells (Figure 8e), suggesting that it might be through regulating HIF-1*α* protein expression that p27 achieved its inhibitory effect in arsenite-induced cell transformation. The overall HIF-1*α* expression at the cellular level was detected using immunocytochemistry (Figure 8f). Therefore, we came to our final conclusion, as delineated in Figure 8f, that by maintaining PHLPP expression, p27 blocked the activation of oncogenic Ras/Raf/MEK/ERK pathway in response to both acute and chronic arsenite exposure, led to inhibition of S6 activation in a p90RSK-dependent manner and consequently attenuated protein translation of HIF-1*α*, a key transcription factor involved in chronic arsenite-induced cell transformation.

## Discussion

Although a variety of research studies has shown that p27 performs cyclin-CDK-independent functions such as mediation of apoptosis, cytoskeleton rearrangements and transcriptional regulation,^23, 24, 37, 38, 39, 40, 41, 42, 43, 44, 45, 46, 47^ the effect of p27 on modulation of protein translation remains largely untouched up to now. Protein translation is a temporally and spatially well-recognized cellular event, and the deregulation of this process contributes greatly to tumor development.^48^ In our current studies, we investigated the role of p27 in arsenite-induced protein translation and its biological significance, as well as the underlying mechanisms. Our findings clearly showed that the depletion of p27 expression by knockdown and knockout approaches resulted in an obvious elevation of S6 phosphorylation, indicating that loss of p27 leads to abnormal activation of the key molecule involved in the specific protein translation, such as HIF-1*α*.

By tracking the upstream pathways responsible for S6 phosphorylation, we found that neither p70S6K nor AKT was involved based on the findings that knocking down p70S6K and loss of the kinase activity of AKT by introducing dominant-negative mutations, K197M and T308/S473A, did not alter S6 phosphorylation. In contrast, blockage of Ras/Raf/MER/ERK pathway by transfecting dominant-negative Ras (S17N) and ERK1/2 (K71/52R) into p27−/−(Δ51) cells attenuated S6 phosphorylation, suggesting that p27 negatively controlled S6 activation by inhibiting the Ras/Raf/MER/ERK/p90RSK pathway. The mechanistic investigation further revealed that p27 maintained PHLPP protein expression, which hampered Ras activation. PHLPP is a newly identified family of Ser/Thr PP and catalyzes the dephosphorylation of a conserved regulatory motif, the hydrophobic motif, on the AGC kinases of AKT, PKC and p70S6K, as well as an inhibitory site on the kinase Mst1, to inhibit cellular proliferation and induce apoptosis.^49^ Therefore, PHLPP is generally regarded as a novel tumor suppressor, and the frequent deletion of PHLPP is observed in human cancers.^49^ The recent findings further showed that PHLPP possessed a novel function in inhibiting Ras activation.^32, 33^ Distinct from the mechanisms of GAPs, PHLPP binds to the nucleotide-free Ras, which occurs during Ras switching from GTP-bound form to GDP-bound form, and in turn abrogates Ras binding with GTP, further leading to repression of downstream ERK pathway activation.^32, 33^ PHLPP expression can be regulated at the translational level by miR-190 in a p50-dependent manner;^50, 51^ however, p27 might control PHLPP expression in an miR-190-independent manner (Zhang and Huang *et al.*, unpublished data).

A malignant tumor develops across time, going through the stages of initiation, promotion and progression.^52^ To observe the tumor induction by chemical carcinogens reproducibly and quantitatively, an *in vitro* assay called cell transformation was developed to detect the conversion of normal cells to tumor cells in culture. Cell transformation is a complex process that involves many transcription factors and the genes that they regulate.^53^ Arsenite induces cell transformation of various types of cells to a more malignant phenotype at relatively low doses that represent the exposure concentrations in nature environment.^53, 54^ It has been demonstrated that the hypoxia signaling pathway is affected by exposure to arsenic, and chronic exposure to low-dose arsenite induced HIF-1*α* protein expression, which contributed to tumor angiogenesis.^55^ We took advantage of the normal mouse epidermal Cl41 cell model to further exploit the potential of p27 in the inhibition of arsenite-induced cell transformation. By tracing the gain of transformation potential using soft agar monthly, we successfully caught a time window during which control transfectants did not manifest transformation characteristic while shRNA p27 transfectants already gained the anchorage-independent growth properties after 3 months of arsenite treatment. By checking the expression profiles of arsenite chronically treated cells, we were excited to reveal that the verified signal pathway that was negatively modulated by p27 in response to acute high-dose arsenite was exactly reproduced in the transformed cells. Therefore, it is concluded that the attenuation of Ras/Raf/MEK/ERK/p90RSK pathway by p27 might result in the abrogation of S6-dependent HIF-1*α* protein translation and arsenite-induced cell transformation as a result.

In summary, from a novel point of view, our findings provided strong evidence for the suppressive effect of p27 in the process of HIF-1*α* protein synthesis in arsenite responses by regulating the PHLPP-Ras-ERK-p90RSK-S6 axis, which may help to upgrade our understanding about the tumor-suppressive functions of p27.

## Materials and Methods

### Cell culture

The 3T3 protocol-immortalized p27 wild-type (p27+/+) and p27-deficient (p27−/−) MEFs,^28, 29^ as well as their stable transfectants, were maintained at 37 °C in 5% CO_2_ incubator with Dulbecco's modified Eagle's medium (DMEM) supplemented with 10% fetal bovine serum (FBS), 2 mM L-glutamine and 25 *μ*g/ml of gentamicin. Mouse epidermal Cl41 cells were cultured in MEM with 5% FBS. The cultures were dissociated with trypsin and transferred to a new 75-cm^2^ culture flask (Fisher, Pittsburgh, PA, USA) two times a week. FBS was purchased from Nova-Tech (Grand Island, NE, USA). Rapamycin was purchased from Calbiochem-EMD Millipore (Darmstadt, Germany).

### Constructs and transfection

shRNA constructs against mouse p27 and S6 were purchased from Open Biosystems (Thermo Fisher Scientific, Huntsville, AL, USA). shRNA construct against mouse HIF-1*α* was purchased from Sigma-Aldrich (St. Louis, MO, USA). mEGFP-HRas S17N was purchased from Addgene (Boston, MA, USA). HA-PHLPP was kindly provided by Dr. Tianyan Gao (Markey Cancer Center and Department of Molecular and Cellular Biochemistry, University of Kentucky, Lexington, KY, USA). Dominant-negative EKR1/2 plasmids (HA-tagged rat ERK1 K71R and HA-tagged rat ERK2 K52R) were as published previously.^56^ GFP-p27 was from Dr. Gustavo Baldassarre (Division of Experimental Oncology, Centro di Riferimento Oncologico, National Cancer Institute, Aviano, Italy). HA-S6 K235/236A construct was kindly provided by Dr. Randal S Tibbetts (Department of Human Oncology, Program in Molecular and Cellular Pharmacology, University of Wisconsin School of Medicine and Public Health, Madison, WI, USA).^18^ All the transfectants were used as a mass pool rather than individual clones.

### Clonogenic survival assay

The cells were plated in triplicate into 100-mm culture dish at the density of 500 cells per dish and cultured for 2 weeks. Cells were fixed by ice-cold methanol and stained with Giemsa solution, and the number of colonies was counted manually and presented as mean±S.D. (*n*=3).

### Reverse transcription-polymerase chain reaction

Total RNA was extracted from the cells using Trizol reagent (Invitrogen, Carlsbad, CA, USA). Total cDNAs were synthesized by ThermoScript RT-PCR system (Invitrogen). The mRNA amount presented in the cells was measured by semiquantitative reverse transcription-polymerase chain reaction (RT-PCR). The primers used were as follows: for mouse *hif-1a* (product 518 bp), 5′-TGGCAAGCATCCTGTACTGT-3′ and 5′-TCCATGTGACCATGAGGAAA-3′ for mouse *gapdh* (product 283 bp), 5′-TGCAGTGGCAAAGTGGAGATT-3′ and 5′-TTTTGGCTCCACCCTTCAAGT-3′ for mouse *β-actin* (product 287 bp), 5′-GACGATGATATTGCCGCACT-3′ and 5′-GATACCACGCTTGCTCTGAG-3′. The PCR products were separated on 2% agarose gels, stained with EB and scanned for images under UV light. The results were displayed with an Alpha Innotech SP image system (Alpha Innotech Corporation, San Leandro, CA, USA). Real-time PCR was carried out by 7900HT Fast Real-time PCR system (Applied Biosystems, Carlsbad, CA, USA). The primer used for mouse *hif-1a* (product 198 bp) were 5′-TCAAGTCAGCAACGTGGAAG-3′ and 5′-TATCGAGGCTGTGTCGACTG-5′. The initial activation was performed at 95 °C for 15 min, and followed by 40 cycles (denaturation at 95 °C for 15 s, annealing at 55 °C for 30 s and extension at 70 °C for 30 s). The data were analyzed as described in the previous publication.^57^

### Ras activity assay

Ras activity kit (Millipore, Billerica, MA, USA) detects the interaction between GTP-bound Ras (from cell lysate) and GST-c-Raf RBD (aa 1–149) that is coated with agarose beads. In brief, the cells were homogenized on ice in 1 × Mg^2+^ Lysis/Wash Buffer. The lysate was centrifuged at 15000 × *g* for 10 min at 4 °C to remove cellular debris. Equal amounts of lysate were incubated for 30 min at 4 °C with GST-c-Raf RBD agarose beads. The beads were then washed three times with ice-cold lysis buffer, and boiled for 5 min at 95 °C. The pull-down of active Ras was analyzed by western blotting.^58^

### Immunohistochemistry

The arsenic chronically exposed cells as indicated were grown on sterile chamber slides (Thermo Fisher Scientific) overnight at 37 ºC. Cells were fixed in 3.7% formaldehyde for 10 min, permeabilized in 0.2% Triton X-100 for 10 min and blocked in 5% BSA for 1 h at 25 °C. The HIF-1*α* antibody (H1alpha67, Abcam, Cambridge, MA, USA) was used at 1 : 200 dilution in 5% BSA containing 0.2% Triton X-100 at 4 °C overnight. An HRP-conjugated polymer-based detection reagent for mouse IgG (Cell Signaling Technology) was used as the secondary antibody at room temperature for 30 min. Reactions were visualized with DAB (Vector Laboratories, Burlingame, CA, USA) and the images were captured by Leica DM 2000 LED microscope (Buffalo Grove, IL, USA).

### [^35^S]methionine pulse assays

Cells were exposed to 20 *μ*M of arsenite for 9 h and then incubated with methionine-cysteine-free DMEM (Gibco-BRL) containing 2% dialyzed fetal calf serum (Gibco-BRL) and 50 *μ*M of MG132 for 1 h. The cells were then incubated with 2% FBS methionine-cysteine-free DMEM containing ^35^S-labeled methionine/cysteine (250 *μ*Ci per dish, Trans 35S-Label; PerkinElmer, Waltham, MA, USA) for the time periods indicated. The cells were extracted with a lysis buffer (Cell Signaling Technology, Beverly, MA, USA) containing a mixture of complete protein inhibitor (Roche, Indianapolis, IN, USA) on ice. Five hundred micrograms of total lysates were incubated with anti-HIF-1*α* antibody and protein A/G agarose beads (Santa Cruz Biotechnology, CA, USA) overnight at 4 °C. The immunoprecipitated samples were washed with the cell lysis buffer five times, heated at 100 °C for 5 min and subjected to SDS-polyacrylamide gel (PAGE) analysis. ^35^S-labeled HIF-1*α* protein was autographed with the PhosphorImager (Molecular Dynamics, Typhoon, Pittsburgh, PA, USA).

### Western blotting assay

The cells were washed two times with ice-cold PBS and collected with the cell lysis buffer (10 mM Tris-HCl, pH 7.4, 1% SDS and 1 mM Na_3_VO_4_). The cell extracts were sonicated, denatured by heating at 100 °C for 5 min and quantified with a Dc protein assay kit (Bio-Rad, Hercules, CA, USA). Equal aliquots of cell extracts were separated on SDS-PAGE. The proteins were then transferred to PVDF membranes (Bio-Rad), blocked and probed with one of the antibodies against phospho-S6 S235/236, phospho-ERK T202/Y204, phospho-MEK1/2 S217/221, phospho-c-Raf S388, S289/296/301, phospho-p90RSK S359/363, S380, S573, total-ERK, total-MEK, total-c-Raf, total-p90RSK, total-S6, Ras and GAPDH (Cell Signaling Technology), p27 for C-terminal (Abcam), HIF-1*α* and PHLPP (Bethyl Laboratories Inc., Montgomery, TX, USA), p27 for N-terminal and HA (Santa Cruz Biotechnology) or *β*-actin (Sigma). Primary antibody-bound proteins were detected by using an alkaline phosphatase-linked secondary antibody and an ECF Western Blotting System (Amersham, Piscataway, NJ, USA).

### Statistical analysis

The significance of the difference between the treated and untreated groups was determined with the Wilcoxon's rank-sum test. The results are expressed as mean±S.D.

## Abbreviations

CHX: cycloheximide
DMEM: Dulbecco's modified Eagle's medium
FBS: fetal bovine serum
HIF-1: hypoxia-inducible factor-1
HRE: hypoxia-responsive element
MEF: mouse embryonic fibroblast
p90RSK: 90 kDa ribosomal S6 kinase
PHLPP: pleckstrin homology domain leucine-rich repeat protein phosphatase
RT-PCR: reverse transcription-polymerase chain reaction
